# The roles of encoding, consolidation, and retrieval in problem solving in mild cognitive impairment and Alzheimer's disease

**DOI:** 10.1002/alz70857_105140

**Published:** 2025-12-25

**Authors:** Lily M Wilkin

**Affiliations:** ^1^ California State University, Fresno, Fresno, CA, USA

## Abstract

**Background:**

Memory encoding, consolidation, and retrieval are impaired in the Alzheimer's disease (AD) process. These cognitive abilities can play important roles in real world problem solving, such as being able to determine the correct amount of medication to take based on instructions. The Item‐Specific Deficit Approach (ISDA) is a psychometrically sound method that separates encoding, consolidation, and retrieval on list learning tests. The current study examined the roles of these memory subprocesses, as assessed by the ISDA, in everyday problem solving in healthy older controls, mild cognitive impairment (MCI), and Alzheimer's disease (AD).

**Method:**

Participants included 58 healthy controls, 65 persons with MCI, and 18 individuals with AD. The ISDA was used to derive indices of encoding, consolidation, and retrieval deficits from performances on the California Verbal Learning Test‐II (CVLT‐II). Real world problem solving was assessed using the Everyday Problems Test (EPT).

**Result:**

A hierarchical regression was conducted to determine whether encoding, consolidation, and retrieval predicted EPT scores, separately for each diagnostic group. Findings indicated that consolidation predicted EPT performances in MCI. The memory subprocesses did not predict EPT performances in healthy older controls or in those with AD.

**Conclusion:**

Our findings demonstrate that memory consolidation specifically plays an important role in the ability to solve problems in the real world in those with MCI. This was not found in healthy older controls or in those with AD. These results suggest the use of the ISDA is beneficial for determining the specific memory subprocesses that will contribute to difficulties with solving problems in everyday life in MCI.

